# Genomic Characterization of Antibiotic Resistant *Escherichia coli* Isolated From Domestic Chickens in Pakistan

**DOI:** 10.3389/fmicb.2019.03052

**Published:** 2020-01-17

**Authors:** Muhammad Rafique, Robert F. Potter, Aura Ferreiro, Meghan A. Wallace, Abdul Rahim, Akbar Ali Malik, Naila Siddique, Muhammad Athar Abbas, Alaric W. D’Souza, Carey-Ann D. Burnham, Naeem Ali, Gautam Dantas

**Affiliations:** ^1^Department of Microbiology, Quaid-I-Azam University, Islamabad, Pakistan; ^2^The Edison Family Center for Genome Sciences & Systems Biology, Washington University School of Medicine, St. Louis, MO, United States; ^3^Department of Biomedical Engineering, Washington University in St. Louis, St. Louis, MO, United States; ^4^Department of Pathology and Immunology, Washington University School of Medicine, St. Louis, MO, United States; ^5^National Reference Laboratory for Poultry Diseases, National Agricultural Research Centre, Islamabad, Pakistan; ^6^Department of Animal Genomics and Biotechnology, PARC Institute of Advanced Studies in Agriculture, National Agricultural Research Centre, Islamabad, Pakistan; ^7^Department of Molecular Microbiology, Washington University School of Medicine, St. Louis, MO, United States; ^8^Department of Pediatrics, Washington University School of Medicine, St. Louis, MO, United States

**Keywords:** antibiotic resistance, genomics, *E. coli*, poultry, global health

## Abstract

Poultry husbandry is important for the economic health of Pakistan, but the Pakistani poultry industry is negatively impacted by infections from *Escherichia coli*. We performed Illumina whole genome sequencing on 92 *E. coli* isolates obtained from the livers of deceased chickens originating in five Pakistani geographical regions. Our analysis indicates that the isolates are predominantly from the B1 and A clade and harbor a diverse number of antibiotic resistance and virulence genes, with no linkage between phylogeny and antibiotic resistance gene presence but some association between phylogeny and virulence gene and SNP presence for the B1 and E phylogroups. The colistin resistance gene *mcr-1* and the quinolone resistance gene *qnrS1* were both found in 13/92 isolates. Alarmingly, 82/92 of the *E. coli* strains characterized in this study are multidrug resistant with 100% (92/92) resistance to lincomycin, 81.5% (75/92) to streptomycin, 79.3% (73/92) to ampicillin and 66.3% (61/92) to ciprofloxacin. These results provide a high-resolution analysis of poultry-associated *E. coli* isolates in an area with a high endemic burden of antibiotic resistance. Surveillance of antibiotic resistance in poultry associated *E. coli* isolates is an important pillar of the One Health concept to integrate analysis of potential pathogens in human, animal, and environmental niches.

## Introduction

The poultry industry is an important component of Pakistan’s gross domestic product (Hussain et al., 2015). However, the Pakistani poultry industry faces several environmental conditions that threaten continued economic output, livestock health, and human health, including pathogen *Escherichia coli* (Hussain et al., 2015; Manges, 2016). *E. coli* is particularly relevant to human health, as *E. coli* can cause a diverse array of infections, exist as a gut commensal, and is often antibiotic resistant through horizontally acquired antibiotic resistance genes (ARGs) (Croxen and Finlay, 2009). In poultry, these infections often manifest as colibacillosis, which can lead to many health abnormalities, often resulting in chicken death (Dho-Moulin and Fairbrother, 1999). Numerous studies have investigated pathogenicity of avian associated *E. coli* isolates and identified factors such as *iss* (serum survival), *ibeA* (cell invasion), *sitA* (iron acquisition), and *iroN* (iron acquisition), which are associated with increased virulence in both chickens and humans (Mellata et al., 2003; Sabri et al., 2006; Cieza et al., 2015; Sarowska et al., 2019). Unlike the strong associations identified between human uropathogenic *E. coli* and the B2 *E. coli* phylogroup, previous analysis of European *E. coli* from birds has not identified genetic signatures strongly associated with any single phylogroup (Cordoni et al., 2016).

Given the economic importance of the domestic poultry industry to Pakistan and the frequent identification of ARGs emanating from the central Asian region (CDDEP, 2017), a comparative phenotypic and genotypic analysis of multidrug-resistant *E. coli* from poultry infections throughout Pakistan is warranted. Total consumption of antimicrobials by chickens in Asia is expected to increase by 129% between 2010 and 2030, driven in large part by increasing chicken consumption in India and Pakistan concomitant with population growth and poor antibiotic stewardship (Hussain et al., 2015; Van Boeckel et al., 2015). Although policies have recently been introduced to encourage restricted antibiotic use in food animals in Pakistan, challenges to regulation and surveillance remain such that accurate estimates of antibiotic usage in poultry rearing are elusive (Rahman and Mohsin, 2019). The types of antibiotics used might be inferred from the multi-drug resistance profiles identified in *E. coli* isolates by previous studies, alarmingly with high prevalence to antibiotics of clinical importance (Idrees et al., 2011; Akhtar et al., 2016; Azam et al., 2019). However, these studies have been limited by the geographic range of their isolates and lack of whole genome sequencing and analysis.

Because antibiotic resistance transmission rates in poultry-associated bacteria may be high in Pakistan due to poultry rearing conditions, constant antibiotic selection, and horizontal gene transfer, it is important to closely monitor the landscape of antibiotic resistance in the Pakistan poultry industry as a whole using genomic methods. To address the knowledge gap on ARG burden in *E. coli* from chickens in Pakistan, we collected *E. coli* isolates from the livers of chicken in five regions of Pakistan and performed Illumina whole-genome sequencing. We then analyzed the population structure, identified relevant gene presence, and assayed for phenotypic antibiotic resistance in these isolates, with the primary goals of identifying any associations between virulence or resistance determinants and phylogroup, or between phylogroup and geographic region.

## Materials and Methods

### *E. coli* Cohort

A total of 1,219 liver samples from culled layer and broiler chickens that had poor birth growth and reduced appetite but not otherwise symptomatic for colibacillosis were collected from the National Reference Laboratory for Poultry Diseases in Pakistan via federal and provincial sentinel surveillance laboratories under a national surveillance program from 2015 to 2017. As the National Reference Laboratory for Poultry Disease does not handle live animals for experimentation, ethics approval was not sought or obtained for this study. The chickens originated in the Pakistani provinces of Balochistan, Sindh, Punjab, Khyber Pakhtunkhwa, and the federally administered Islamabad Capital Territory. Whole liver samples were cultured in nutrient broth (Sigma-Aldrich, St. Louis, MO, United States) at 37°C for 24 h. A loopful of the overnight culture was then plated onto eosin-methylene blue agar and grown at 37°C for 24 h. Suspected *E. coli* isolates were identified using the API 20E assay (bioMérieux, Durham, NC, United States), and glycerol stocks were generated. Frozen cultures were sent to Washington University in St. Louis for further analysis. Isolates were plated onto blood agar and *E. coli* identification was confirmed with the MALDI-TOF VITEK MS IVD v2.3.3 (bioMérieux, Durham, NC, United States) mass spectrometry system (Richter et al., 2013).

### Illumina Whole-Genome Sequencing

Frozen stocks of *E. coli* isolates were plated onto blood agar using four-quadrant streaking and ∼10 morphologically similar colonies from the fourth quadrant were used as input for the QIAamp BiOstic Bacteremia DNA Kit (Qiagen, Germantown, MD, United States). A total of 0.5 ng of genomic DNA per isolate was used to create sequencing libraries with the Nextera Kit (Illumina, San Diego, CA, United States) (Baym et al., 2015). The libraries were pooled together at equimolar concentrations and sequenced on a NextSeq 500 to obtain 25–183X coverage of each genome with 2 bp × 150 bp reads. The reads were demultiplexed by barcode and Illumina adaptors and contaminating sequences were removed with Trimmomatic v.38 (Bolger et al., 2014) and Deconseq v.4.3 (Schmieder and Edwards, 2011), respectively. The processed reads were used to construct *de novo* assemblies of each genome with SPAdes v3.13.0 (Bankevich et al., 2012). The assembly metrics of the *scaffolds.fasta* files were assessed with QUAST v4.5 (Gurevich et al., 2013) and open reading frames identified with Prokka v1.12 (Seemann, 2014). A total of 92 genomes with less than 300 contigs were chosen for downstream genomic and phenotypic analysis (Supplementary Table S1).

### *In silico* Analysis

To obtain phylogroup information for each *E. coli* genome, we gathered 11 publicly available genomes from known *E. coli* phylogroups and identified open reading frames using Prokka (Supplementary Table S2) (Schreiber et al., 2017; Hutton et al., 2018). The *gff* files from Prokka for the phylogroup reference strains and the genomes sequenced in this study were used as input for Roary v3.12.0 to construct a core-genome alignment of the 2,755 core-genes with PRANK v1.0 (Loytynoja, 2014; Page et al., 2015). The core-genome alignment file was converted into an approximate maximum likelihood tree with FastTree v2.1.10 and the resulting newick file was uploaded to iToL^1^ (Letunic and Bork, 2007; Price et al., 2010). In parallel, we identified *in silico* antibiotic resistance determinants for acquired antimicrobial genes using ResFinder v4.0 and for *E. coli* single nucleotide polymorphisms with PointFinder v4.0 (Kleinheinz et al., 2014; Zankari et al., 2017). Additionally, we identified known virulence genes with VirulenceFinder v1.5 and Enterobacteriaceae plasmid replicons with PlasmidFinder v4.0 (Carattoli et al., 2014; Kleinheinz et al., 2014). Hypergeometric tests were used to determine the significant enrichment of isolate groups within phylogenetic clades or dendrogram clusters, with Bonferroni correction for multiple hypothesis testing.

### Antibiotic Susceptibility Testing

Phenotypic antibiotic resistance assessment was performed using a protocol similar to those previously described for various *Enterobacteriaceae* on a variety of antibiotics relevant to human and veterinary use (Potter et al., 2018a, b). Briefly, the phenotypic antibiotic resistance of *E. coli* isolates was assayed through growth on Mueller-Hinton agar in the presence of antibiotic-laden Kirby-Bauer disks in accordance with Clinical Laboratory and Standards Institute requirements for human derived isolates and also for veterinary derived isolates (Supplementary Tables S3, S4) (CLSI, 2018). The interpretation of zone of clearance was used to create a heatmap with hierarchical clustering for each isolate in pheatmap (R studio). ComASP^TM^ (Liofilchem) was used exactly in concordance with manufacturer’s instructions to quantify colistin resistance in isolates with identified *in silico* resistance determinants. Disk diffusion values for ceftriaxone/ceftiofur and ciprofloxacin/norfloxacin were plotted as XY coordinates in Prism v8.0 and linear regression analysis was performed using default conditions to quantify the *R*^2^ value.

## Results

**FIGURE 1:**
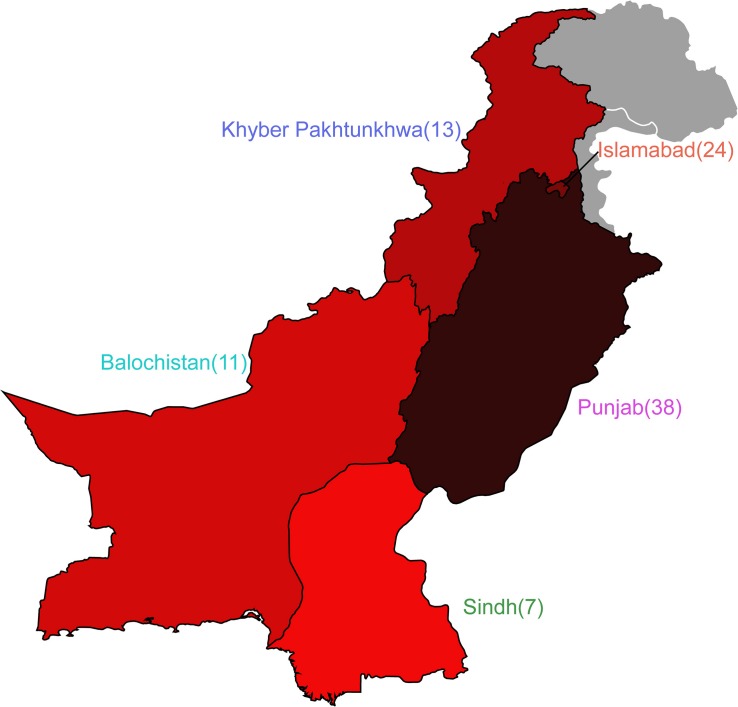
*Escherichia coli* cohort originates from five regions in Pakistan. Map depicting the four provinces of Pakistan and the Islamabad Capital Territory from which isolates were obtained. Number of isolates are shown adjacent to the provinces, and colors correspond to isolate prevalence.

**FIGURE 2:**
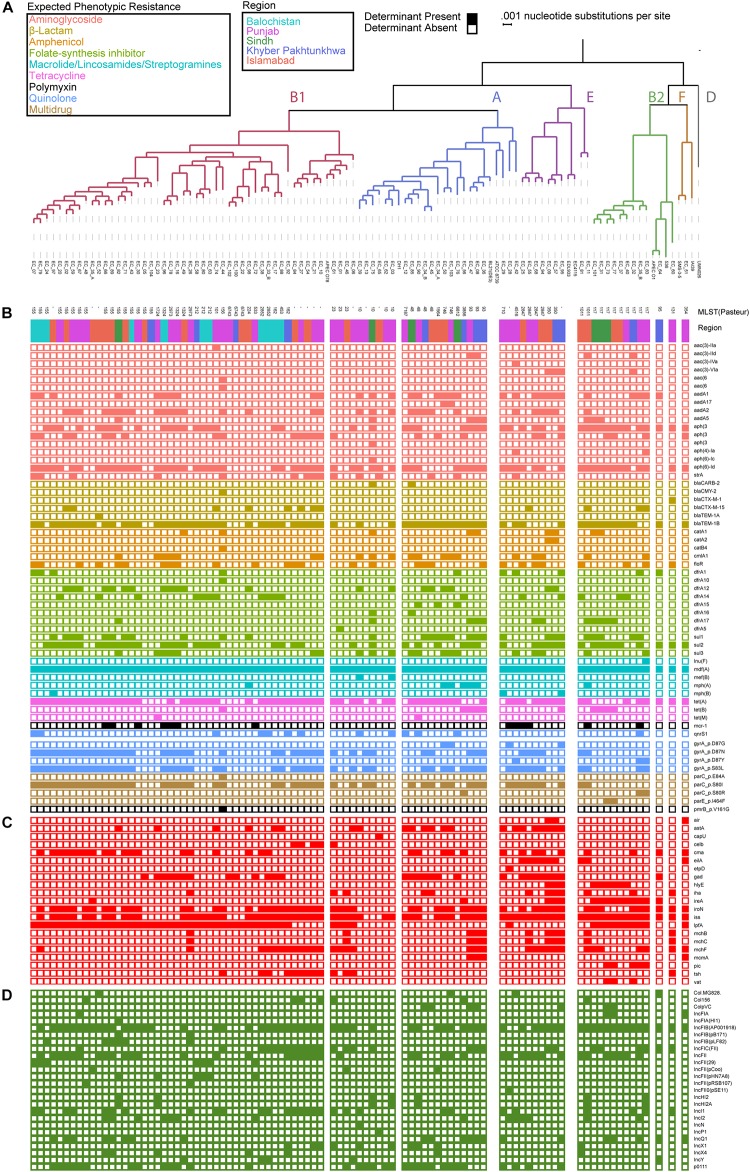
*Escherichia coli* genomes are predominantly in B1 and A phylogroup with a mosaic of antibiotic resistance determinants and virulence genes. **(A)** Population structure of the *E. coli* cohort depicting the phylogroup, MLST, and region obtained. Presence absence of antibiotic resistance determinants **(B)**, virulence genes **(C)**, and plasmid replicons **(D)** identified using ResFinder, PointFinder, VirulenceFinder, and PlasmidFinder on the cohort.

Of the 92 genomes that were used for full genomic and phenotypic analysis, 41.3% (38/92) originated in Punjab and 26% (24/92) came from Islamabad. The remainder of the isolates originated from Khyber Pakhtunkhwa (14%; 13/92), Balochistan (12%; 11/92), and Sindh (7%; 7/92) (Figure 1). Phylogenetic reconstruction of the similarity between the *E. coli* within our cohort and with known phylogroup strains indicates that there is not a clear association between phylogroup and geographic region, although we note isolates from Balochistan fell exclusively in the B1 Clade (Figure 2). Overall, 53.2% (49/92) of the cohort are in the B1 Clade. The rest of the isolates are in Clade A (22/92), B2 (11/92), E (9/92), and F (1/92). The most abundant sequence types were ST115 (16/92) in B1 and ST117 (9/92) in phylogroup B2 (Figure 2A).

**FIGURE 3:**
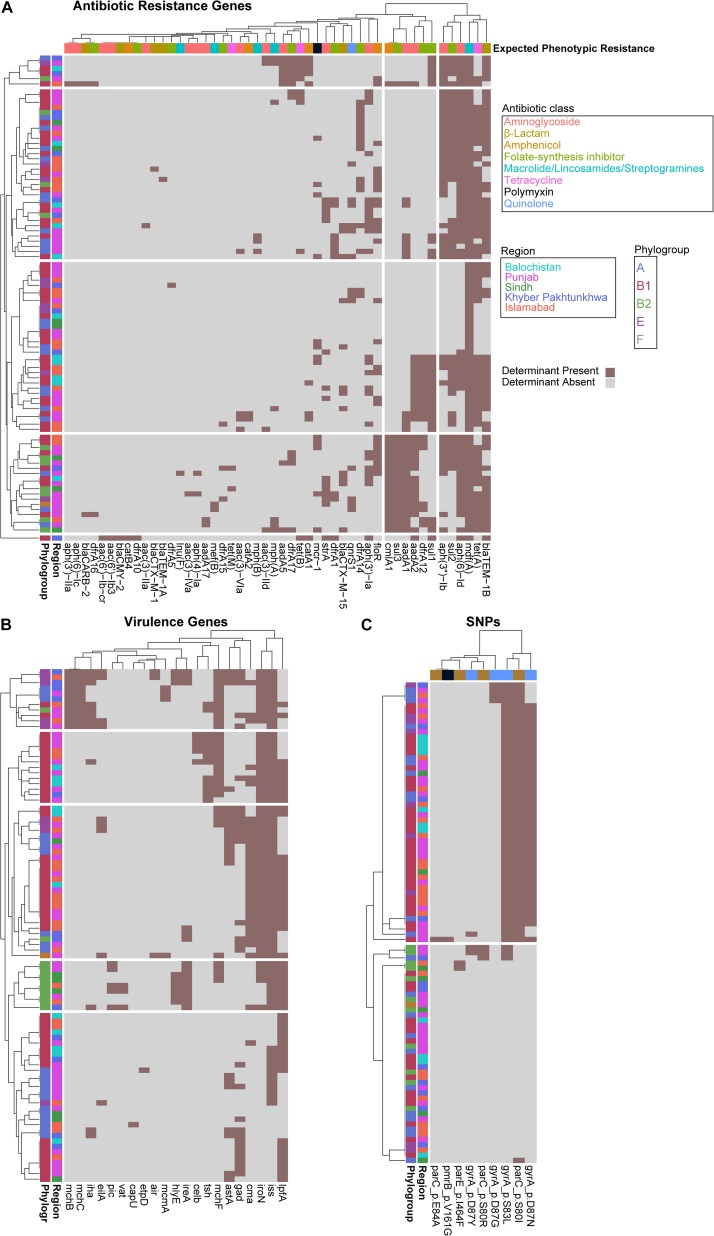
*Escherichia coli* phylogroups segregate better by virulence gene presence than ARG or SNP presence. Heatmaps depicting isolates as rows and ARGs **(A)**, virulence genes **(B)**, or SNPs **(C)** as columns. Rows and columns are hierarchically clustered by Euclidian distance. Region, phylogroup, or expected phenotypic resistance conferred by ARGs are portrayed as metadata, as indicated.

To identify a genotypic basis for phenotypic antibiotic resistance, following Illumina whole-genome sequencing, we applied ResFinder to identify acquired ARGs and PointFinder to locate relevant SNP resistance determinants in the assembled genomes. Consistent with previous reports on genomic analysis of *E. coli* isolates, we identified a mosaic of antibiotic resistant determinants and virulence genes within our cohort with no clear association between ARG composition, phylogroup, and region source, other than the identification of all isolates from Balochistan as members of the B1 phylogroup (*p* < 0.01, hypergeometric test) (Figure 2A). In total, we identified 49 unique ARGs and 5 previously validated antibiotic resistance-conferring SNPs (Figure 2B) (Zankari et al., 2017). The median number of ARGs per isolate was 7 and the median prevalence for each ARG was 8. We found that 17/49 of the ARGs are predicted to have activity against aminoglycosides, 11/49 against folate-synthesis inhibitors, 6/49 against β-lactams, 5/49 against amphenicols, and 5/49 against lincosamides. In addition, we identified 3 *tet* ARGs (76 isolates), and the quinolone resistance gene *qnrS1* (13 isolates). PointFinder identified amino acid changes in GyrA (D87G 4/92 isolates, D87N 44/92 isolates, D87Y 3/92 isolates, and S83L 53/92 isolates), ParC (E84A 1/92 isolates, S80I 51/92 isolates, S80R 3/92 isolates), ParE (I464F 2/92 isolates), and PmrB (V161G 1/92 isolates). The aminoglycoside ARGs include representatives of the *aac*, *aadA*, and *aph* families. Notably, no known carbapenem resistance genes were identified within this *E. coli* cohort. The most prevalent β-lactamase was *bla*_TEM–__1__B_ (found in 66/92 isolates). The class A β-lactamases *bla*_CTX–M–__15_ and *bla*_CTX–M–__1_ were found in 1/92 and 12/92 isolates. The only non-class A β-lactamase present was *bla*_CMY–__2_, which was found in a single isolate. The most conserved gene, *mdf(A)*, conferring multidrug resistance, was found in 92/92 of the *E. coli* isolates, indicating that it is a core-gene within this cohort (Edgar and Bibi, 1997). *mdf(A)* was the sole ARG in 7/92 of the isolates. Despite this conservation, the other lincosamide ARGs, *lnu(F)* (1/92), *mef(B)* (2/92), *mph(A)* (7/92), and *mph(B)* (3/92) were at or below the median ARG prevalence. The colistin ARG *mcr-1* was found in 13/92 of the *E. coli* isolates. The prevalence of *mcr-1* was much higher than SNPs in genes associated with colistin resistance as only 1/92 isolates contained the V161G amino acid change in PmrB. When hierarchically clustered on ARG presence (*k* = 5), no segregation of isolates by region or phylogroup is observed, except for an enrichment of B2 isolates (*p* < 0.01) in a cluster characterized by a high ARG load that includes *bla*_TEM–__1__B_, *tet(A)*, and *mdf(A)*, as do most of the isolates, but also *aadA2*, *aadA1*, *sul3*, and *cmlA1* (Figure 3A). When hierarchically clustered on antibiotic resistance-conferring SNPs, the isolates are segregated into two major clades, one with a preponderance of SNPs in *gyrA* and *parC*, and a clade of isolates most of which carry no SNPs (Figure 3C). B2 isolates are enriched in the latter clade (*p* < 0.001).

We applied VirulenceFinder on the sequenced *E. coli* cohort to annotate genes putatively involved in poultry infections (Figure 2C). We identified 21 virulence genes and found that the median number of virulence genes per isolate was four. Only 2/92 of the isolates had no known virulence genes identified. Consistent with their previously identified roles in *E. coli* virulence, the serum survival gene *iss* was found in 78.2% (72/92) of the isolates, the iron acquisition gene *iroN* was in 60.9% (56/92) of the cohort, and the long polar fimbriae gene *lpfA* was in 59.8% (55/92) of the cohort. When hierarchically clustered on virulence gene presence (*k* = 5), we again observed no segregation of the isolates by geographic region (Figure 3B). However, the B2 and E isolates were each enriched in their own clusters (*p* < 1E-9 and *p* < 0.01, respectively) characterized by different virulence gene profiles. While the larger B1 phylogroup was more distributed across the clusters, there was one cluster that was exclusively comprised of B1 isolates (*p* < 0.001). Together these data indicate that the phylogroups segregate better by virulence gene presence than ARG presence; the lack of segregation by geographic region suggests that chicken-borne *E. coli* strains are readily transmitted across Pakistan.

We applied PlasmidFinder using the Enterobacteriaceae database on our *E. coli* cohort to identify known plasmid replicons (Figure 2D). 90/92 isolates had plasmid replicons identified, with a maximum of 7 in EC_10, EC_44, and EC_67, and a median of 4.26 different replicons were identified, among which members of the IncF plasmid replicon family were the most prevalent (12/26). IncFIB(AP001918) and IncFII were the most prevalent among the isolates with 65/92 and 41/92 identified within the cohort, respectively.

**FIGURE 4:**
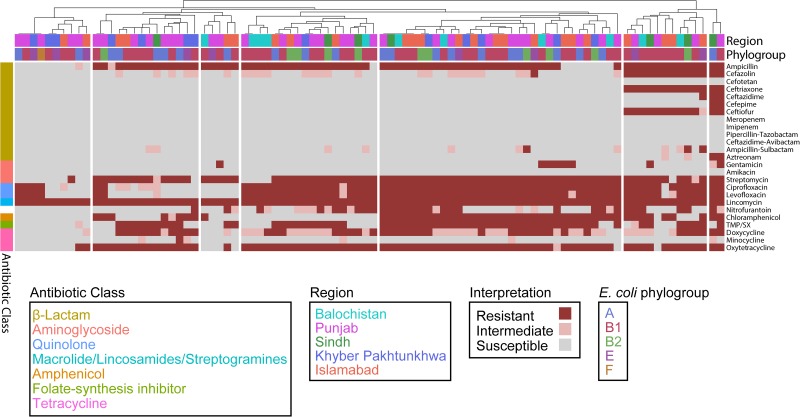
*Escherichia coli* isolates are predominantly (82/92) MDR. Heatmap depicting each isolate as a column and each row as an antibiotic. Columns are hierarchically clustered by Euclidian distance. Region and phylogroup are portrayed as metadata.

To assess the effect of ARG burden on phenotypic antibiotic resistance, we performed antimicrobial susceptibility testing using the Kirby-Bauer Disk Diffusion method and the Clinical Laboratory and Standards Institute (CLSI) interpretative criteria from the M100 (Edition 29) and VET01 (Edition 5) for *Enterobacteriaceae* on a variety of antibiotics relevant for human and veterinary use. Using the definition of multidrug-resistant (MDR) as non-susceptibility to at least one agent in three or more antimicrobial classes, and extensively drug resistant (XDR) as susceptibility to at least one agent in only one or two classes assayed, we found that 82/92 are MDR but only 1/92 are XDR (Figure 4) (Magiorakos et al., 2012). Consistent with the presence of *mdf(A)* in all the genomes, all isolates were resistant to the lincosamide antibiotic lincomycin. Hierarchal clustering on the antibiotics with CLSI interpretive criteria (using 1 for resistant, 0 for intermediate, and −1 for susceptible) with a cluster cutoff just below the second node of the dendrogram resulted in seven clusters (*k* = 7) (Figure 4). The first cluster (from left to right) was characterized by susceptibility to β-lactams. There was widespread resistance to quinolones and the aminoglycoside streptomycin across all clusters, while the two rightmost clusters were characterized by additional resistance to the β-lactams cefazolin, ceftriaxone, and ceftiofur. There was no significant association between any cluster and any region or phylogroup, unlike the associations detected between clusters formed on genetic features and phylogroup (Figure 3). Given problems using disk diffusion testing for colistin resistance, we performed a broth minimum inhibitory concentration (MIC) assay using the ComASP^TM^ colistin test on all *mcr-1* positive isolates, the *pmrB* SNP isolate 55, quality control strain *E. coli* ATCC 25922, and the mcr-1 positive *E. coli* AR Bank #0350 from the CDC and FDA Antibiotic Resistance Isolate Bank (Table 1). All *mcr-1* positive isolates had MIC values of 4 or 8 μg/mL but the *pmrB* SNP isolate 55 had an MIC of 0.25 μg/mL. This cohort demonstrated 100% *in vitro* susceptibility to meropenem, imipenem, cefotetan, piperacillin-tazobactam, and amikacin. We found strong association between phenotypic resistance within the 3^rd^ generation cephalosporin ceftriaxone and ceftiofur (*R*^2^ = 0.9004) and quinolones ciprofloxacin and norfloxacin (*R*^2^ = 0.8897) (Supplementary Figure S1). One isolate (EC_44) was discordant for the 3^rd^ generation cephalosporins and tested as resistant to ceftriaxone but intermediate to the veterinary antibiotic ceftiofur and contained *bla*_CMY–__2_ and *bla*_TEM–__1__B_. While there are not CLSI interpretative criteria for norfloxacin, one isolate (EC_72) without any identified quinolone resistance determinants tested as ciprofloxacin susceptible but had a comparatively low disk diffusion radius to norfloxacin.

**TABLE 1:** Colistin susceptibility testing results.

**Isolate**	**Putative resistance determinant**	**Colistin MIC (μg/mL)**
EC_07	*mcr-1*	8
EC_10	*mcr-1*	4
EC_12	*mcr-1*	4
EC_25	*mcr-1*	4
EC_38	*mcr-1*	4
EC_44	*mcr-1*	8
EC_50	*mcr-1*	4
EC_51	*mcr-1*	4
EC_55	*pmrB* mutation	0.25
EC_62	*mcr-1*	4
EC_67	*mcr-1*	4
EC_68	*mcr-1*	4
EC_79	*mcr-1*	4
EC_98	*mcr-1*	8
*E. coli* ATCC 25922	None	0.5
*E. coli* AR Bank #0350	*mcr-1*	4

## Discussion

Antibiotics are widely used in the poultry industry of developing nations for the prevention of disease and growth promotion (CDDEP, 2017). Despite growing concern over the selective pressure this contributes to antimicrobial resistance and the potential for this to expand the reservoir for the dissemination of MDR pathogens, global annual consumption of antimicrobials by food animals is expected to increase by 67% between 2010 and 2030 (Van Boeckel et al., 2015). To our knowledge, this study represents the first use of next-generation sequencing and bioinformatics techniques in combination with phenotypic susceptibility testing to assess the extent of antibiotic resistance in poultry *E. coli* isolates from multiple provinces of Pakistan.

Given that chicken infections by *E. coli* can be explained by atypical environmental conditions leading to increased stress-related immunosuppression, some groups argue that the concept of avian pathogenic *E. coli* has no strong basis (Hoerr, 2010; Shini et al., 2010; Collingwood et al., 2014). This is consistent with our analysis that did not find any common genetic signature associated with chicken-borne *E. coli* isolates (Zhu Ge et al., 2014; Paudel et al., 2016). To understand if the chicken-borne *E. coli* isolates within our cohort have an identifiable population structure, we applied Roary and FastTree to the core-genome of the *E. coli* cohort and a selection of reference phylogroup strains to construct a phylogenetic tree. Our results indicate that a majority of the isolates belong to the B1 or A clades. It is possible that a greater extent of genetic signatures related to phylogroup may be identified with the more stringent criteria of sequencing isolates derived from active chicken infections, and not just birds with failure to grow. These findings are similar to those describing *E. coli* from bovine mastitis in Ireland, which also predominantly yielded B1 and A clade genomes (Keane, 2016). An analysis of avian associated *E. coli* isolates from several countries in Europe found variation within each country but that A1 and B2 phylogroups were the most prevalent, indicating that on a continental scale geographic location may affect *E. coli* background (Cordoni et al., 2016). A broader analysis of European avian associated *E. coli* isolates came to similar results with B2 and A as the dominant phylogroups (Mora et al., 2013). Since there was not a control chicken gut arm of our study, it is possible that the B1 and A clade isolates we found are also found as commensals in a greater proportion of the chickens. We found that the phylogroups are represented evenly in the different regions of Pakistan, except for the finding that all Balochistan isolates were B1. This may be simply due to the low sample size from that region (11 isolates), but the fact that we did observe multiple phylogroups in the similarly sized isolate sets from Sindh and Khyber Pakhtunkhwa, and that most of Balochistan is geographically separated from the rest of Pakistan by the Sulaiman and Brahui ranges (southern offshoots of the Hindu Kush Himalayan Region), suggest the possibility that phylogenetic segregation of chicken-associated *E. coli* at the scale of Pakistan’s geography may be detected in a larger collection of isolates. The B2 isolates repeatedly segregated when the isolates were hierarchically clustered by ARG, virulence gene, or SNP presence, as well as phenotypic resistance, reflecting the longer branch lengths of these isolates in the phylogeny in Figure 2A compared to most of the cohort. The phylogroups segregated better by virulence gene presence than ARG presence, possibly reflecting the increased mobility of ARGs. We found that IncFIB plasmid replicons were the most prevalent in our cohort. An analysis of *E. coli* in the United States found that IncFIB plasmids were significantly more present in *E. coli* suspected as avian pathogens or from retail poultry than compared to human and avian commensals (Johnson et al., 2007, 2008).

Of greatest concern from our analysis of the ARG content within the cohort was the presence of the mobilizable colistin ARG, *mcr-1*, in 14% (13/92) of the isolates, all of which displayed phenotypic resistance to colistin. Initially discovered in *E. coli* obtained from swine farms in China, it has since been identified in a wider cohort of bacteria including in a cohort of avian associated *E. coli* isolates from Egypt and China, although at a much lower prevalence of 1% (12/1220 isolates) (Liu et al., 2016; Barbieri et al., 2017). A wider analysis of *E. coli* from chickens in 13 Chinese provinces found that *mcr-1* was detected in 4% (58/1136) of the genomes (Yang et al., 2017), while an analysis of 100 *E. coli* chicken isolates from Faisalabad, Pakistan found an *mcr-1* prevalence of 8% (Lv et al., 2018). An *E. coli* isolate from a poultry farm in Tunisia was found to harbor both *bla*_CMY–__2_ and *mcr-1* and exhibited resistance to several other antibiotic classes (Maamar et al., 2018). *bla*_CMY–__2_ was identified once in our cohort and that isolate did not have *mcr-1* but it did have the *pmrB* V161G mutation which has been previously shown to confer colistin resistance, but did not in our study (Delannoy et al., 2017). Similar to previous analyses of *E. coli* isolates from poultry, *iss* was the most common virulence gene identified in our cohort (Keane, 2016). Deletion of *iss* from an isolate significantly perturbed *E. coli* growth in serum (Huja et al., 2015).

We found that 82/92 isolates are MDR using the definition of MDR as non-susceptibility to at least one agent in three or more antimicrobial classes (Magiorakos et al., 2012). This result is similar to analysis of avian associated *E. coli* isolates from Nepal, which determined that 94% (47/50) of their cohort was MDR (Subedi et al., 2018); and from Hebei, China which had 100% (87/87) MDR (Li et al., 2015). One analysis of avian associated *E. coli* from the Punjab region in Pakistan found almost universal resistance to ampicillin (98.6%) while we found 79.3% (73/92) (Azam et al., 2019). In Egypt, one analysis of 116 avian associated *E. coli* isolates found 100% (116/116) resistance to ampicillin (Awad et al., 2016). A study of retail poultry products from the United States found that *E. coli* originating from turkey products had an ampicillin resistance rate (62%) higher than *E. coli* originating from chicken products (20%) (Davis et al., 2018). Thankfully a number of clinically relevant antibiotics (meropenem, imipenem, cefotetan, piperacillin-tazobactam, and amikacin) had no phenotypic resistance observed. This result is consistent with a previous report of environmental *E. coli* isolates from Japan, described by the authors as harboring a virulence gene profile similar to isolates associated with avian infections, which were 100% susceptible to carbapenems and aminoglycosides (Hayashi et al., 2019).

A limitation of this study was that with short Illumina reads, we were unable to unequivocally implicate ARGs or virulence genes as present on mobilizable plasmids. As we did not sequence isolates originating from the intestinal contents of these chickens, we are not able to discuss the similarity and differences between the liver-borne isolates and those from commensal sites. Additionally, we do not have access to a chicken model of infection to demonstrate links between comparative pathogenicity of the strains and their virulence gene mosaic. Given the limitation of clinical breakpoints some isolates that possess genetic determinants of resistance may test phenotypically susceptible. Since we tested multiple antibiotics in the same antibiotic classes there is not always a clear relation between phenotypic resistance and genotypic presence of a resistant determinant. As we used disk diffusion to assess antimicrobial susceptibility instead of quantitative broth microdilution (except in cases of *mcr-1* positive isolates) we are not able to report MIC values. In summation, we assembled a cohort of chicken associated *E. coli* isolates obtained from multiple provinces in Pakistan. Genomic analysis of these isolates identified that most of the cohort are in the B1 and A clades and harbor a mosaic of ARGs and virulence genes which may complicate treatment in a human infection.

## Data Availability Statement

All genomes sequenced in this study have been uploaded to the NCBI WGS database associated with BioProject PRJNA522294.

## Author Contributions

MR and RP sequenced the isolates, performed the *in silico* analysis, and wrote the manuscript. AF performed the *in silico* analysis and helped to write the manuscript. MW carried out the culture work and susceptibility testing. AR, AA, NS, and MA collected and stored the samples. AD helped to write the manuscript and generate the figures. C-AB, NA, and GD devised the study.

## Conflict of Interest

The authors declare that the research was conducted in the absence of any commercial or financial relationships that could be construed as a potential conflict of interest.

## Footnotes

1 https://itol.embl.de/

## Supplementary Material

FIGURE S1 Strong concordance between disk diffusion relationships. Scatter plots depicting the disk diffusion relationship for the 3^rd^ generation cephalosporins ceftriaxone and ceftiofur **(A)** and quinolones ciprofloxacin and norfloxacin **(B)**.

TABLE S1 Genomes analyzed in this study.

TABLE S2 Publicly available genomes used in this study.

TABLE S3 Antimicrobial susceptibility testing information.

TABLE S4 Full antimicrobial susceptibility testing results.
