# Supplementary figures and images for: Genomic Characterization of Antibiotic Resistant Escherichia coli Isolated From Domestic Chickens in Pakistan
Source: Front Microbiol. 2020 Jan 17;10:3052. doi: 10.3389/fmicb.2019.03052 (PMC6978674; doi:10.3389/fmicb.2019.03052)

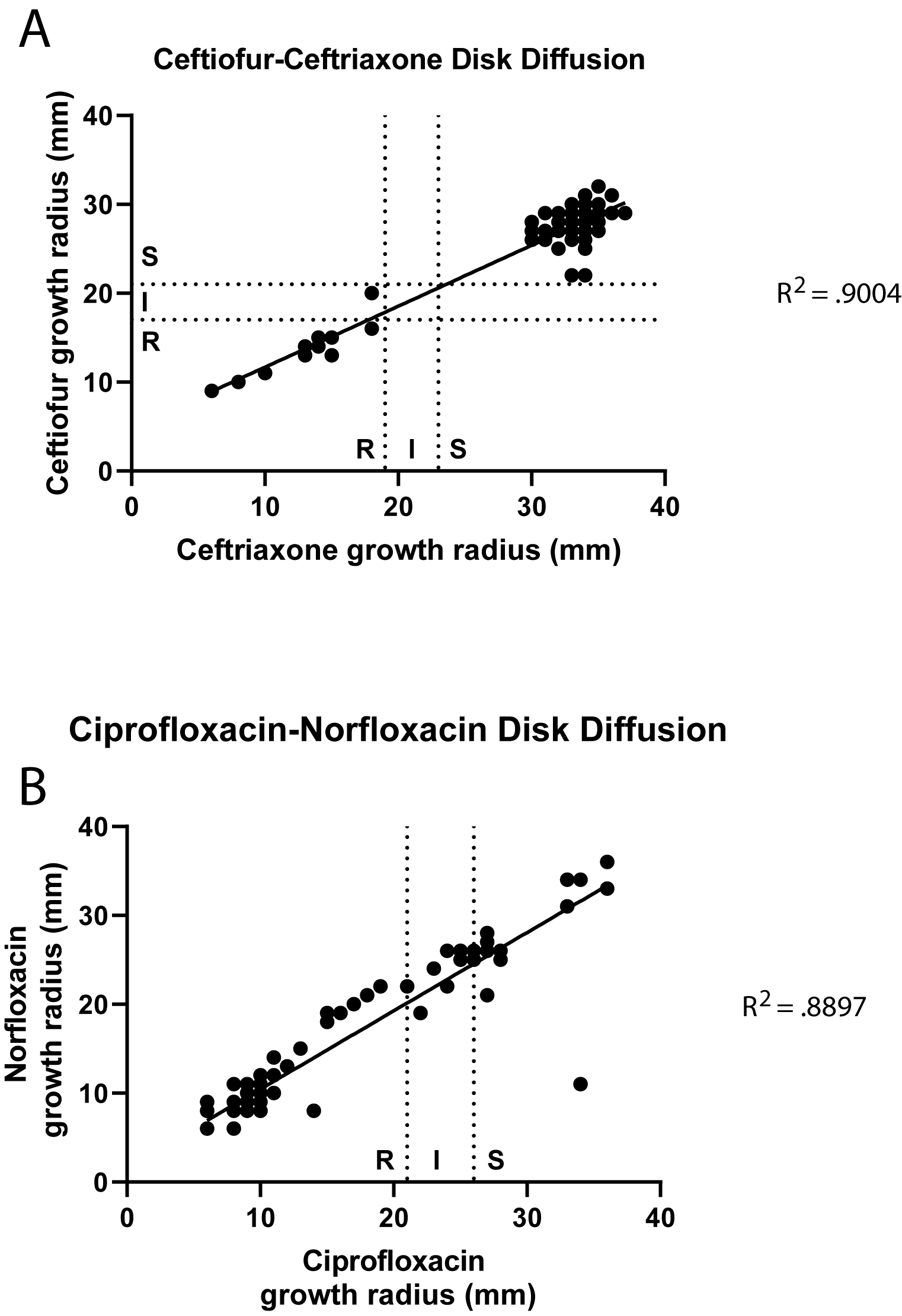

Supplement: FIGURE S1 — Strong concordance between disk diffusion relationships. Scatter plots depicting the disk diffusion relationship for the 3rd generation cephalosporins ceftriaxone and ceftiofur (A) and quinolones ciprofloxacin and norfloxacin (B). [file Image_1.TIF]
